# One-stage efficacy of single tract minimally invasive ECIRS in the improved prone frog split-leg position for staghorn stones

**DOI:** 10.1186/s12894-022-01003-w

**Published:** 2022-04-06

**Authors:** Changyi Liu, Biqiong Zheng, Jinfeng Wen, Houping Mao, Tao Jiang, Qin Chen, Wenwei Chen, Hua Zhang, Yanfeng He, Rui Gao

**Affiliations:** 1 Department of Urology, The First Affiliated Hospital of Fujian Medical University, No. 20, Chazhong Rd., Taijiang District, Fuzhou, Fujian China; 2 Department of Anesthesiology, The First Affiliated Hospital of Fujian Medical University, Fuzhou, China

**Keywords:** Single tract, Minimally invasive, Endoscopic combined intrarenal surgery, Percutaneous nephroscopy, Stone free rate, Improved prone frog split-leg position, Staghorn stones

## Abstract

**Objective:**

To explore the feasibility, safety, and effectiveness of single tract minimally invasive endoscopic combined intrarenal surgery (stmECIRS) in the improved prone frog split-leg position for staghorn stones.

**Method:**

A total of 83 patients with staghorn stones were retrospectively reviewed between January 2018 and June 2021. According to surgical procedure and position, patients were divided into a group of single tract minimally invasive percutaneous nephroscopy (stmPNL) in the prone position and a group of stmECIRS in the improved prone frog split-leg position (turned to the prone position after preset the flexible ureteroscope sheath in lithotomy position, meanwhile, bend both hips and knees to be frog abduction). Demographic characteristics, laboratory tests, stone characters, surgical information, stone-free rate (SFR), and perioperative complications were observed and analyzed.

**Results:**

There were no significant differences in demographic characteristics, changes level of Scr and Hb, stone size, radiation density, length of hospital stay, and operation time between the two groups. One-stage SFR in the stmECIRS group was significantly higher than that in the stmPNL group (84.4% vs. 57.9%) (*P* = 0.007), only 2 patients required blood transfusion after surgery (*P* = 0.862), and other postoperative complications were not statistically significant (*P* = 0.345).

**Conclusions:**

StmECIRS in improved prone frog split-leg position has a higher one-stage SFR than stmPNL for staghorn renal stones, and without complications increased, which is a safe, efficient and feasible treatment.

## Introduction

Staghorn calculi is a refractory disease, which seriously affects the treatment experience and economic burden of patients [1]. According to the European Association of Urology (EAU) guidelines, percutaneous nephrolithotripsy (PNL) is recommended as the standard treatment for staghorn calculi [2]. However, the high stone-free rate (SFR) of PNL often needs to establish multiple access tracts, and the increased number of tracts is the risk factor for postoperative complications, especially bleeding [3, 4]. Minimally invasive PNL (mPNL) use the size of 16F to 20F tract sheath [5], which has advantages of lower postoperative bleeding rate and similar SFR [6, 7], but a disadvantage of lower intraoperative therapeutic efficiency [8]. With the rapid development of endoscopic equipment, flexible ureteroscope (FURS) has been accepted by most urologists to treat renal stones < 2 cm [2, 9], Even for some stones ≥ 2 cm [10, 11]. However, it is difficult and time-consuming to remove the fragment of stones, large stones may need several courses to be completely removed [12]. Therefore, FURS is not recommended as a first-line choice for the treatment of staghorn calculi.

Endoscopic combined intrarenal surgery (ECIRS) is a new technique that can perform PNL and FURS simultaneously, also can reduce the number of access tracts [13]. Related studies have confirmed that the use of single tract minimally invasive ECIRS (stmECIRS) for staghorn calculi has a high SFR and few postoperative complications [14–16]. ECIRS commonly uses the Galdakao-modified supine Valdivia (GMSV) position and the prone split-leg position [17, 18]. However, the GMSV position has some disadvantages, including the collapse of the pyelocaliceal system, difficulties of renal puncture, and smaller space for instrument manipulation, especially in obese patients [19]. Urologists are more familiar with the prone position, but in the prone split-leg position, the learning curve is relatively high to find the ureteral orifice and place the flexible ureteroscope sheath by using FURS in the prone position, particularly in men with ureteral stricture [16, 20], and the actual operation space of the perineal area is small.

This study had improved the prone split-leg position to the prone frog split-leg position, and the ureteroscope sheath was indwelled in the lithotomy position and then change position. Our study aims to explore the feasibility, safety, and efficacy of stmECIRS for staghorn stones under the modified prone frog split-leg position, and to provide more experience and clues for the selection of clinical treatment of staghorn stones.

## Materials and methods

### Patients

We retrospectively reviewed and analyzed 83 patients with staghorn stones who underwent single tract minimally invasive percutaneous nephrolithotripsy (stmPNL) or stmECIRS between January 2018 and June 2021, at the First Affiliated Hospital of Fujian Medical University. The stone branch that occupies each renal calyces (≥ 80% renal pelvis and calyceal volume) was defined as a complete staghorn stone. Stone branch < 80% renal pelvis and calyceal volume were defined as partial staghorn calculi [21]. Inclusion criteria: age ≥ 18 years; the presence of staghorn calculi was confirmed by KUB or CT and the maximum diameter of the stones was ≥ 3 cm; met the indications of PNL in the guidelines of EAU and AUA. Exclusion criteria: ureteral stricture that could not be solved in one stage; patients with abnormal renal anatomy, coagulation dysfunction, solitary kidney, severe urinary tract infection or tuberculosis and severe cardiopulmonary dysfunction.

### Preoperative preparation

We collected clinical information from electric medical records included sex, age, BMI, preoperative and postoperative laboratory results of hemoglobin (Hb), serum creatinine (Scr), and urine culture. The size, location and radiation density of stones were determined by the abdominal plain film (KUB), ultrasonography (US), and computed tomography (CT). The stone size was defined as the maximum diameter of the largest stone. The antibiotics were adjusted according to the preoperative urine culture results. If the urine culture result was negative, patients were given the third-generation cephalosporin antibiotics for 2–3 days before the operation and 4–5 days after the operation. The characteristics of patients and stones were summarized in Table 1. Operation time was defined as the period from the beginning of disinfection to the closure of the wound. Intraoperative ultrasound was mainly used to puncture and determine the location, size and quantity of stones. X-ray fluoroscopy was only used to further confirm whether the puncture needle was successfully placed in the renal pelvis and calyces, and to assist in judging the position, size, and quantity of residual stones after the completion of lithotripsy. The time of fluoroscopy was short, hence the fluoroscopy time was not recorded separately during the operation.

**Table 1:** Demographic and stone characteristic

Characteristic	stmECIRS (n = 45)	stmPNL (n = 38)	P value
Age (years)	54.64 ± 11.58	55.13 ± 11.22	0.847
Gender			0.426
Male	31	23	
Female	14	15	
BMI (kg/m^2^)	23.84 ± 3.49	22.44 ± 2.96	0.054
Hb (g/L)	137.96 ± 13.61	131.89 ± 21.92	0.144
Scr(umol/L)	74.4 (62.4, 82.9)^b^	80.1 (68.1, 94.1)^b^	0.204^a^
Stone side			0.062
Left	27	15	
Right	18	23	
Stone size (mm)	48.7 (41.7, 67.0)^b^	43.1 (35.5, 67.3)^b^	0.128^a^
Mean CT density (HU)	1299.53 ± 282.75	1275.92 ± 345.16	0.733
No. of staghorn calculi			0.368
Complete	13	32	
Partial	10	28	

Data are reported as numbers and percentages or as mean ± SD, as appropriate

StmPNL Group: Single tract minimally invasive percutaneous nephroscopy in prone position was performed for the patients in the prone position

StmECIRS Group: Single tract minimally invasive endoscopic combined intrarenal surgery was performed in the improved prone frog split-leg position

BMI: Body mass index; CT: computed tomography; HU: hounsfifield units

^a^Mann–Whitney U test was used when normal distribution was not obeyed

^b^Non-normally distributed continuous variables were represented by the median (quartile)

### Surgical procedure

#### stmPNL

After successful anesthesia, the Wolf ureteroscope (F8/F9.8) was inserted into the target ureter in the lithotomy position to explore and exclude foreign bodies and severe stricture. A 7F ureteral catheter (Shanghai Shangyi Kangou Medical Equipment Co., LTD., Shanghai, China) was retrogradely placed at the ureteropelvic junction of the target ureter to prepare “artificial hydronephrosis”. Then turn to the prone position and elevate the kidney area of the affected side. The target renal calyces were punctured under the dual guidance of ultrasound and X-ray. After that, used sequential fascial dilators and a matching 20F working sheath (Kangyibo Science and Technology Development Co., Ltd., Shenzhen, China) to establish a single percutaneous renal tract. Then, the Wolf ureteroscope (F8/F9.8) was inserted through the passage into the renal calyces to locate stones, followed mainly using pneumatic trajectory (EMSS.ACH-1260Nyon, SwissLithoClast ®LCM21, Swiss) and partly using 550 μm holmium laser fiber (SRM-H3, Shanghai Reicorn Laser Technology Co., Ltd., Shanghai, China) to shatter the stones.At the end of the operation, a 7F double-J stent (AngiomedGmbH&CO.Medizintechnik,Karlsruhe,Germany) and a 18F nephrostomy tube (Kangyibo) were routinely indwelled.

#### stmECIRS

**Fig. 1:**
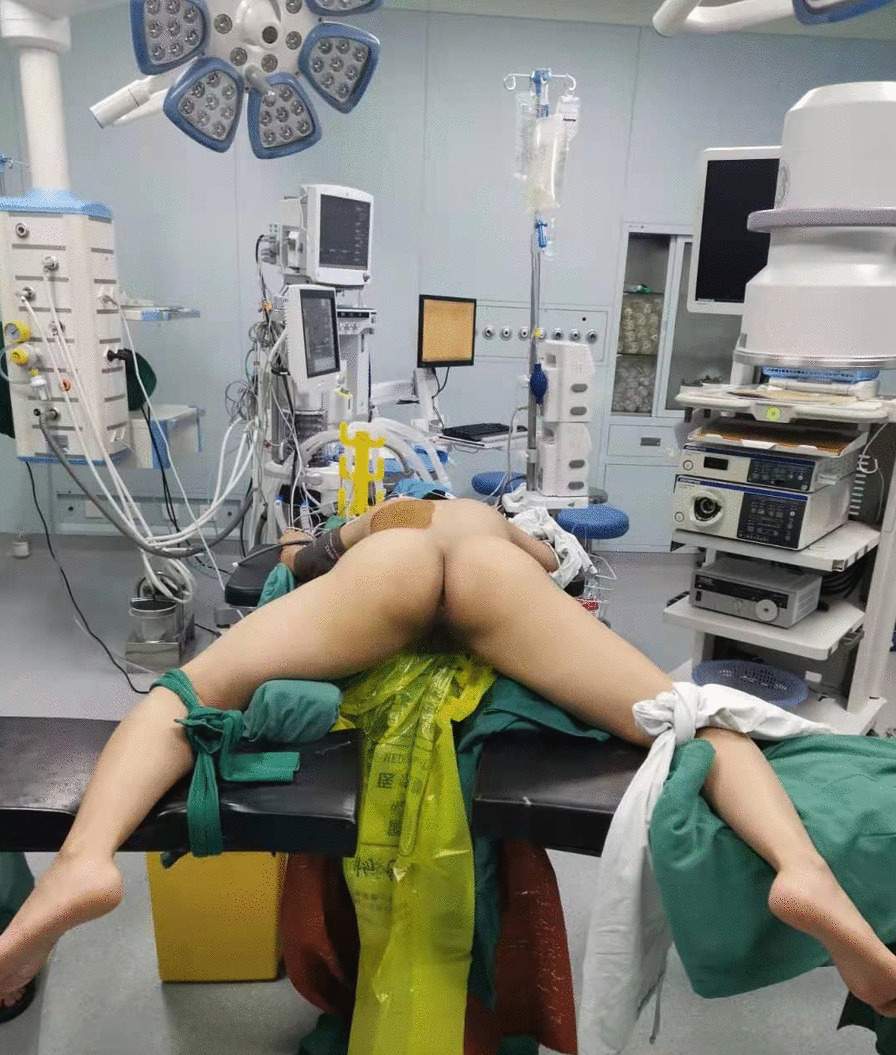
The improved prone frog split-leg position. After preset the flexible ureteroscope sheath into the target ureter in the traditional lithotomy position, turn to prone position, at the same time, bend both hips and knees to be frog abduction, which should not exceed the physiological curvature. The soft postural support pad should be placed on the front wall of both thighs to prevent hanging, and the lower legs should be fixed with a fixing strap

**Fig. 2:**
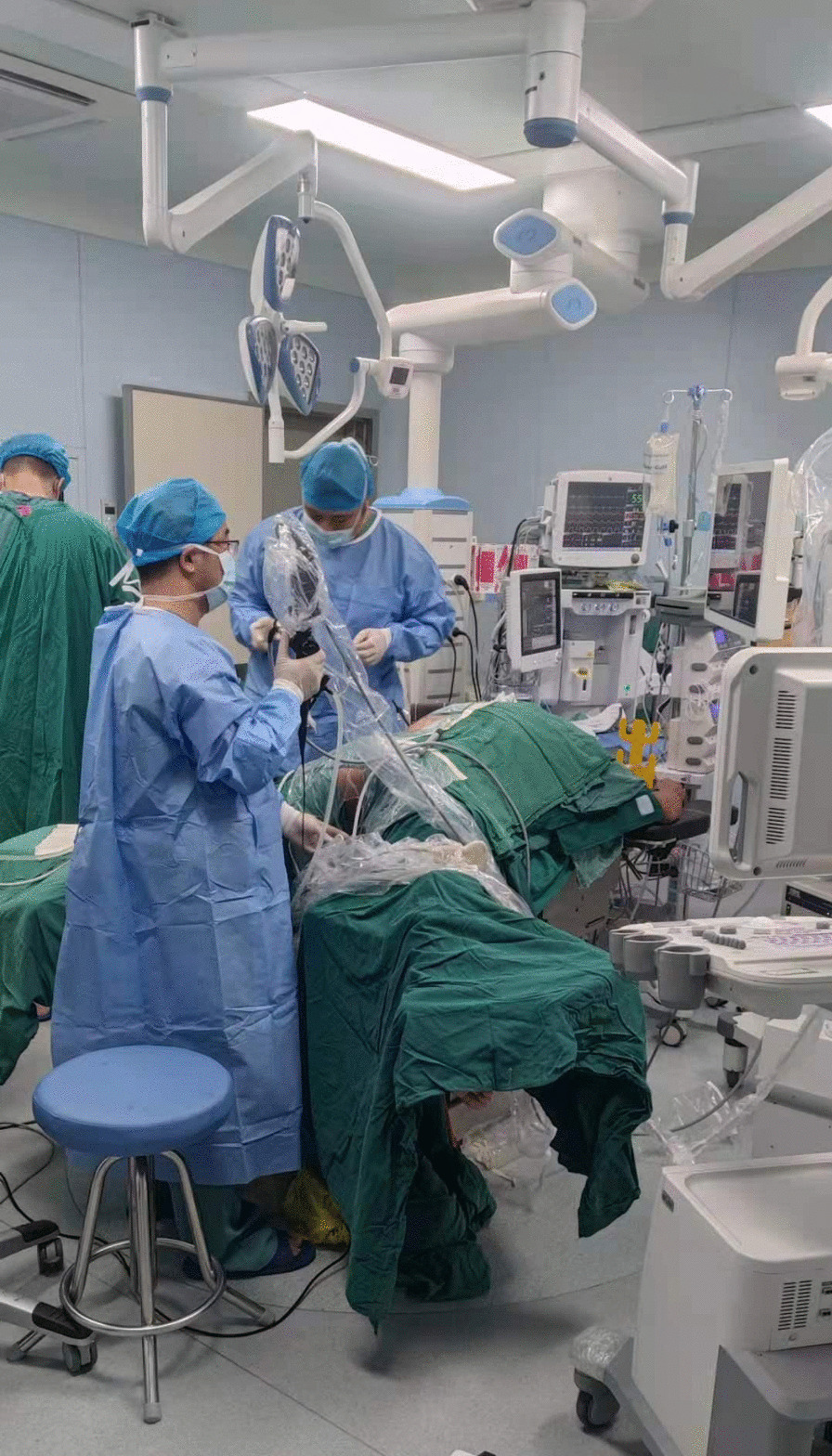
Single tract minimally invasive endoscopic combined intrarenal surgery (stmECIRS) in the improved prone frog split-leg position for staghorn stones. One urologist performed the anterograde single tract minimally invasive percutaneous nephroscopy (stmPNL), another one performed the retrograde flexible ureteroscope (FURS)

First, we used the same method as above to explore the target ureter in the lithotomy position. If there was no abnormality, a guide wire (BostonScientific, Shanghai, China) was placed through the Wolf ureteroscope (F8/F9.8). Then, an X-ray confirmed that the flexible ureteroscope sheath (F12/F14, Cook Medical, Indiana, USA) was inserted into the ureteropelvic junction via the guide wire. An extension tube was placed into the sheath to prepare “artificial hydronephrosis”, and sterile gloves were fully wrapped around the exposed part of the sheath. Subsequently, turn to the prone position, at the same time, bend both hips and knees to be frog abduction, which should not exceed the physiological curvature. The soft postural support pad should be placed on the front wall of both thighs to prevent hanging, and the lower legs should be fixed with a fixing strap (Fig. 1). Carefully disinfect the affected kidney operation area, perineal area and exposed part of flexible ureteroscope sheath. The procedure of stmPCNL puncture was the same as the above method. One urologist used 200 µm fiber optic holmium laser (SRM-H3) to shatter the stones under retrograde flexible ureteroscope. The holmium laser parameters are set as follows: energy 1.0–1.5 J, frequency 10–20 Hz. Another urologist performed anterograde lithotripsy similar to that in stmPNL (Fig. 2). The stone fragments were all removed through percutaneous renal tract. F7 double-J stent tube (Angiomed) and F18 nephrostomy tube (Kangyibo) were indwelled as above.

### Postoperative evaluation

The relevant laboratory indexes were reexamined immediately on the first day after surgery, mainly Hb and Scr. One-stage SFR was determined by KUB or CT 3–5 days after surgery. The postoperative complications of PNL were classified according to Clavien grading system. The stone free status was defined as the diameter of stone fragments < 4 mm [22]. Fever was defined as body temperature > 38.5 °C.

### Statistical method

SPSS 23.0 for windows was used for all data analysis. Kolmogorov–Smirnov test was used to test the normality of continuous variables. The continuous variables of normal distribution were expressed as mean ± standard deviation (SD) and analyzed by independent sample *t*-test. The continuous variables of non-normal distribution are expressed as median and compared by Mann–Whitney U test. The classified variables were expressed as frequency and rate, and analyzed by chi-square test or Fisher exact test. The difference was statistically significant (*P* < 0.05). *P* < 0.05 was considered as statistically significant.

## Results

### Demographic and stone parameters

The demographic parameters and stone characteristics of the two groups are summarized in Table 1. There was no statistically significant difference in preoperative demographic parameters between the two groups. The age, sex, body mass index, Hb and Scr of the two groups were comparable. The average size, side and density of stones between the two groups did not show significant difference.

### Surgical parameters

All patients completed the operation successfully and used 20F working sheath. The specific operation-related parameters are summarized in Table 2. The median operation time of stmPNL group and stmECIRS group were 99.0 (75.8, 150.3) minutes and 105.0 (85.0, 124.0) minutes, respectively, and the operation time of the two groups was similar (*P* = 0.996). The median decrease level of postoperative Hb in stmECIRS group and stmPNL group were 2.4 (1.7, 3.1) g/dL and 2.1 (1.5, 2.7) g/dL (*P* = 0.353), respectively. In this study, changes of renal function were reflected by Scr. No significant difference in the increase level of postoperative Scr between the two groups (*P* = 0.814). The median hospitalization time was 11.5 (10.0, 14.1) days in stmPNL group and 10.0 (8.0, 13.0) days in stmECIRS group (*P* = 0.082).

**Table 2:** Operation-related parameters

Characteristic	stmECIRS	stmPNL	P value
Operative time (min)	105.0 (85.0, 124.0)^b^	99.0 (75.8, 150.3)^b^	0.996^a^
Hb drop (g/dL)	2.4 (1.7, 3.1)^b^	2.1 (1.5, 2.7)^b^	0.353^a^
Scr up (umol/L)	− 2.46 ± 8.97	− 1.89 ± 12.80	0.814
Hospitalization time (d)	10.0 (8.0, 13.0)^b^	11.5 (10.0, 14.1)^b^	0.082^a^
One-stage stone free (%)	38 (84.4)	22 (57.9)	0.007
Complications			0.345
Fever (> 38.5 ℃) (%)	1 (2.2)	0	1.0^c^
Hemorrhage (%)	4 (8.9)	7 (18.4%)	0.202
Blood transfusion (%)	2 (4.4)	2 (5.3)	0.862
Clavien grade			
0 (%)	40 (88.9)	31 (81.6)	0.345
I (%)	3 (6.7)	5 (13.1)	0.318
II (%)	2 (4.4)	2 (5.3)	0.862
≥ III (%)	0	0	

StmPNL Group: single tract minimally invasive percutaneous nephroscopy in prone position was performed for the patients in the prone position

StmECIRS Group: single tract minimally invasive endoscopic combined intrarenal surgery was performed in the improved prone frog split-leg position

Hb: Hemoglobin; Scr: serum creatinine

^a^Mann–Whitney U test was used when normal distribution was not obeyed

^b^Non-normally distributed continuous variables were represented by the median (quartile)

^c^Since the data frequency contains “0”, the result of Fisher's Exact Test in the Chi-square Test was used

### SFR

The preliminary results of imaging examination 3–5 days after surgery showed that the one-stage SFR was 57.9% in stmPNL group and 84.4% in stmECIRS group. The SFR of stmECIRS was significantly higher than that of stmPNL (*p* = 0.007).

### Complications

One patient in stmECIRS group had a body temperature > 38.5 C, which was improved after reduced temperature and continued anti-infective therapy, and got grade I Clavien-Dindo score, while there was no fever in stmPNL group and no sepsis in both groups. 4 cases of postoperative bleeding required treatment in stmECIRS group and 7 cases in stmPNL group. 2 patients in stmECIRS group and 5 patients in stmPNL were stable and got grade I Clavien score after hemostatic treatment, while 2 patients in stmECIRS group and 2 patients in stmPNL group got grade II Clavien score because of blood transfusion. No patient had Clavien score ≥ III. There was no significant difference in the incidence of perioperative complications between the two groups.

## Discussion

The goal of the treatment of staghorn calculi is to remove stones as much as possible, to inhibit stone recurrence, to control urinary tract infection, and to protect the function of the affected kidney to the greatest extent [23]. PNL is still the first-line choice for the treatment of staghorn calculi [2], However, several studies showed that the high stone clearance rate and high bleeding rate of standard tract (24-30F) PNL were accompanied, and the postoperative blood transfusion rate was about 12% to 20% [24–26]. In this study, 20F tract sheath was uesd in both groups, the postoperative blood transfusion rate of stmECIRS group was 4.4%, which was similar to that of stmPCNL group (5.3%). And the rate was significantly lower than that reported in the above study, which was also consistent with the view of related studies that mini-PNL had less postoperative bleeding than traditional PNL [1]. It confirmed that the improved posture of stmECIRS in our study is safe.

ECIRS is a safe and effective emerging technique for the treatment of staghorn calculi [13], which has the advantages of high stone clearance rate and few postoperative complications, and may become a ideal surgical method for staghorn calculi. At present, there are many kinds of postures for ECIRS, and the most popular positions are the GMSV position and the prone split-leg position [3]. Fangzhou Zhao et al. [18] reported that treatment of complex stones with ECIRS in GMSV position and Shuzo Hamamoto et al. [27] reported that treatment of staghorn stones with ECIRS in the prone split-leg position all achieved high SFR (88.1% vs. 81.7%).In this study, stmECIRS group has a more significant SFR than stmPCNL group (84.4% vs. 57.9%, *P* = 0.007), which is also comparable with the results of the above study. It confirmed that the improved posture stmECIRS in our study is effective.

Different from the prone split-leg position in the study of Shuzo Hamamoto et al. [14], which need to use flexible ureteroscope to find the target ureteral orifice directly in the prone position. Our study need to preset the flexible ureteroscope sheath into the target ureter in the traditional lithotomy position, and then change the position to the prone split-leg position for surgery.The reasons to made this improvement lies in: first, looking for the target ureteral orifice in the prone position is an irregular position, which is not in line with the operating habits of most urologists, and the flexible ureteroscope has disadvantages of small field of vision and slow water injection. Its own problems were difficulty in entering the ureteroscope and whether the flexible ureteroscope sheath can be successfully inserted, particularly in male patients [16, 20]. Thereby, the learning curve is higher. On the one hand, preset the flexible ureteroscope sheath in lithotomy position can facilitate surgeon to solve the problems encountered in indwelling sheath, such as annular ureteral stricture, on the other hand, it can also reduce the learning curve of beginners. Second, the operative space in the perineal area in the prone split-leg position is relatively small, and the activity of surgeon may be limited. The prone frog split-leg position in our study can more fully expand the operative space in the perineal area without exceeding the physiological curvature, and increase the surgical comfort of the surgeon. Compared with the prone split-leg position, the GMSV position also has the trouble in difficuly of retrograde indwelling the sheath [28], and urologists are more familiar with PNL in the prone position. The prone position has advantages of shorter puncture distance, wider puncture range, larger operation space and lower migration of the renal area during the operation, surgeons can be more handy during the operation [19, 29].

The writer summarizes some experiences of the new posture in this study: (1) Although it is still necessary to change the position during the operation, it can reduce the difficulty of retrograde insert the ureteroscope and the failure rate of indwelling flexible ureteroscope sheath in the prone position, especially for male patients. (2) It is very necessary to routinely perform ureteroscopy on the affected side before indwelling the flexible ureteroscope sheath. We can observe the conditions of the target ureter and rule out the presence of severe stricture and tortuosity of the ureter which is not suitable for one-stage FURS surgery. (3) CT and KUB films should be fully read before surgery to select the target renal calyces that can reach the most calculi area, and accurate puncture of the renal calyces should be carried out under the guidance of ultrasound and X-ray simutaneously to improve surgical efficiency. (4) The stones only need to be shatter to until that can pass through the calyx neck when used holmium laser fiber lithotripsy under flexible ureteroscope, then, used stone basket to drag the stones into the middle or passage calyx, and the other surgeon fragmented and flushed out the stones by nephroscope, which can reduce the operation time repeatedly.

This study is a single-center retrospective design and has certain limitations. Firstly, the number of cases enrolled was relatively small. Secondly, we chose larger than 3 cm stones for surgery, but patient selection bias may still exist. Although the safety and efficacy of stmECIRS in prone frog split-leg position have been preliminarily discussed, it still needs to be confirmed by a large sample prospective multicenter study.

## Conclusion

StmECIRS in improved prone frog split-leg position is a safe, efficient, and feasible method for staghorn stones, with higher one-stage SFR than stmPNL, and no increase in complications. Compared with the GMSV position and prone split-leg position, the prone frog split-leg position retained the advantages of prone position, at the same time, flexible ureteroccope sheath was preset in lithotomy position, and frog split-leg expanded the operative space, which greatly reduces the difficulty curve for urologists to learn ECIRS technique, and is worth popularizing and applying in clinic.

## Data Availability

The datasets generated during the current study are available from the corresponding author on reasonable request.
